# Food Expenditure and Food Consumption before and during Ramadan in Moroccan Households

**DOI:** 10.1155/2020/8849832

**Published:** 2020-12-08

**Authors:** Imane Barakat, Hamid Chamlal, Sanaa El jamal, Mohammed Elayachi, Rekia Belahsen

**Affiliations:** Laboratory of Biotechnology, Biochemistry and Nutrition, School of Sciences, Chouaib Doukkali University, El Jadida 24000, Morocco

## Abstract

Fasting in the month of Ramadan is a religious, cultural, and social ritual for Muslims. The benefits it is supposed to provide to people who practice it are often impaired by unhealthy lifestyles including diet. The present research aimed to study risky eating behaviors and the variation in food expenditure of the Moroccan population during Ramadan. This study was conducted in 2018 in 340 households in urban and rural localities in the Rabat-Salé-Kenitra region of Morocco. Information on eating habits was collected by a nutritional survey using the method of dietary history; household food expenditure and sociodemographic characteristics were collected by a questionnaire. The average age of the population is 40 ± 14 years; the majority (40%) has a middle standard of living, and the average food expenditure increased by 50% during Ramadan. The food survey showed a significant increase in energy intake (*p* < 0.001), carbohydrate intake (*p* < 0.001), sucrose intake (*p*=0.02), sodium intake (*p* < 0.001), and calcium intake (*p* < 0.001) and a significant decrease in protein intake (*p* < 0.001) and lipid intake (*p* < 0.001), with no significant change in saturated fatty acid intake (*p*=0.86) during Ramadan. These results show that some dietary behaviors adopted during Ramadan could promote the development or worsening of overweight and chronic diseases. These data reveal the importance of nutritional education adapted to this sacred month.

## 1. Introduction

The month of Ramadan is a ritual practiced by Muslims, characterized by a profound change in their lifestyle, in particular, by abstinence from eating and drinking, from sunrise to sunset with a change in activity [1] and sleep schedules [2]. Other implications of this sacred month consist in the variation in household expenditure and food intake affecting their economic and nutritional status, respectively [3]. In fact, a study has revealed an increase in the daily consumption of carbohydrates and proteins and a decrease in that of lipids and saturated fatty acids during Ramadan [4]. Another study reported that daily intake of energy, fat, and protein increases, while carbohydrate intake decreases [5]. Another research, on the contrary, showed a decrease in caloric intake and an increase in saturated fatty acids (SFAs) and simple sugars during Ramadan [6]. Furthermore, it has been reported that calcium is the micronutrient whose intake decreases the most during Ramadan [7] with a variation during this sacred month according to the age of the subjects studied [8, 9]. Certainly, the month of Ramadan has many advantages, in this case, the reduction of food inequality and poverty and solidarity actions [10]. Nevertheless, changes in the eating system and eating habits, in association with an abundance of food, have occurred in recent years and have led to poor eating behaviors adopted during this holy month accompanied by changes in weight, dehydration, or worsening of the health of patients with chronic diseases such as hypercholesterolemia, hypertension, hyperuricemia, kidney and liver disease, and diabetes [11, 12]. In addition, some studies have reported weight loss with or without change in body composition [13], while other studies have reported that body weight and composition remain unchanged [14] and may even increase [15]. The effect of Ramadan eating habits on fasting glucose and serum lipid profiles has also been reported by the literature [16]. Moreover, transient effects of Ramadan fasting on the health of healthy adults have been revealed. These effects may be positive, such as decreased weight and fat, or negative, such as increased low-density lipoproteins (LDLs) and insulin resistance [17]. Similarly, a meta-analysis study found that Ramadan fasting can change weight and certain biochemical parameters in healthy subjects, especially in men [18]. The literature has also revealed the effect of Ramadan fasting on the circadian rhythm of hormones, including leptin, adiponectin, prolactin, insulin, and cortisol, inducing insulin resistance [19].

The purpose of the present research was to study variations in food expenditure and risky eating behaviors during the month of Ramadan in an adult population in the Rabat-Salé-Kenitra region. The importance of this study lies in the identification of harmful eating behaviors adopted by subjects practicing Ramadan fasting, in order to propose appropriate educational prevention measures.

## 2. Methods

### 2.1. Target Population and Sampling Method

The study targeted 340 households in both urban and rural communities, randomly drawn from seven prefectures and provinces in the Rabat-Salé-Kenitra (RSK) region of Morocco, including Kenitra, Khemisset, Rabat, Salé, Sidi Kassem, Sidi Slimane, and Skhirate-Témara. The survey was conducted in 2018, at the households' level visited by dieticians. The questionnaire was completed by one subject per household aged 18 and over practicing fasting.

### 2.2. Questionnaire and Food Survey

The socioeconomic characteristics, in particular, the age, the gender, the area of residence, the standard of living, and food expenditure, were collected using a questionnaire developed by the research team. Food consumption data were collected using the diet history questionnaire [20]. The quantities of food and food preparations consumed were estimated in the study population one week before and one week during the month of Ramadan in 2018, using an iconographic manual [21]. Data collection was carried out by dieticians trained on the questionnaire adopted for this study.

The questionnaire used was adapted and supplemented from our previous studies and the reliable reference literature on diet history questionnaire. The tool was then pretested on a sample of 25 women to verify the understanding of the questions and the reproducibility of the answers to the questions. It is then validated by an expert committee made up of a doctor, physiologist, nutritionist, dietitian, and statistician. Dietary intakes were analyzed to calculate nutritional values, by the Microsoft Bilnut Program version 2.01.

### 2.3. Variables

The characteristics of the study population were described by age, gender, place of residence, and standard of living. The same categorization reported in the literature for the variables age [9] and standard of living is used [22]. Food expenditure is estimated on the basis of the food budget before and during Ramadan. The nutritional intakes determined are the energy intake (Kcal/day), protein (g/day), carbohydrate (g/day), fat (g/day), sucrose (%) of total energy intake, SFA (g/day), sodium (mg/day), and calcium (mg/day). Interest has been focused on these contributions since they are targeted by several public health programs including the National Nutrition Program in Morocco [23] and that the literature has reported their variation during Ramadan.

### 2.4. Statistical Analysis

The statistical analysis of the data was performed by the SPSS (Statistical Package for the Social Sciences) software for Windows, version 21. The sample distribution was studied by the Kolmogorov–Smirnov normality test. This test revealed that the explanatory variables have an asymmetric distribution and hence their median and quartile expression [*Q*1; *Q*3]. Comparison tests were used taking into account the asymmetric distribution of variables; in this case, we used the Kruskal–Wallis test for independent series with more than two groups and the Wilcoxon test for paired series. The statistical significance threshold was set at 5% (*p* ≤ 0.05).

### 2.5. Ethical Considerations

The questionnaire used in this study was validated by a scientific committee of the University Chouaib Doukkali of El Jadida, and the data collection was started after obtaining an authorization from the Regional Health Directorate in the Rabat-Salé-Kenitra region of Morocco. The fundamental ethical principles governing the conduct of research were respected in this study, and free and informed prior consent was obtained from participants.

## 3. Results

### 3.1. Characteristics of the Population

Table 1 shows that 48% of the population studied is over 40 years of age, 70.6% resides in urban areas, and 40% has a middle standard of living. Gender is represented almost equally, 52% for men and 48% for women. The average household food expenditure was 1600 MAD per month before Ramadan and 2500 MAD per month during Ramadan corresponding to a 50% increase in food expenditure during Ramadan compared to other months of the year.

### 3.2. Food Expenditure before and during the Month of Ramadan according to Standard of Living

Table 2 shows that food expenditure increases significantly by standard of living, both before and during Ramadan (*p* < 0.01). However, the rate of variation in these expenditures according to standard of living did not increase significantly during Ramadan (*p*=0.35). This increase is greater in households with a low standard of living (50%) compared to those with medium (39%) and high (40%) standard of living.

### 3.3. Rate of Variation in DNI according to RNI

Table 3 shows that some daily nutritional intakes (DNIs) before and during Ramadan are higher than the recommended nutrient intakes (RNIs) [24, 25], in particular, energy intake, respectively, 12% and 18%; protein intake, 48% and 30%; carbohydrate, 20% and 38%; sucrose, 70% and 80% of total energy intake (TEI); SFA, 40% for both periods; and sodium, 54% and 64%. The lipids are 11% higher before Ramadan and 4% lower during Ramadan compared to the RNI, and the calcium is 40% lower before Ramadan and 33% lower during Ramadan compared to the RNI.

### 3.4. Nutritional Intake Comparison before and during Ramadan

Table 4 shows a significant increase in energy intake (2247 [1895; 2745] vs (in this work, “vs” is used as an abbreviation of versus) 2367 [2040; 2700]; *p* < 0.001), carbohydrate (312 [253; 391] vs 360 [303; 430]; *p* < 0.001), sucrose (17 [12, 21] vs 18 [14, 21]; *p*=0.02), sodium (3688 [2452; 4873] vs 3928 [2772; 5121]; *p* < 0.001), and calcium (478 [377; 623] vs 540 [452–689]; *p* < 0.001) as well as a significant decrease in protein intake (74 [55; 89] vs 65 [60; 75]; *p* < 0.001) and lipid intake (78 [59; 97] vs 67 [58; 85]; *p* < 0.001). However, there was no change in the SFA contribution (28 [22, 37] vs 28 [23, 37]; *p*=0.86) during the holy month compared to the other months of the year.

### 3.5. Nutritional Intake before and during Ramadan according to Age

Table 5 shows that, for the three age groups [18; 25 years], [26; 40 years], and ≥40 years, there is, respectively, a significant increase in energy intake ((2203 [1862; 2699] vs 2328 [2037; 2689]; *p*=0,001), (2178 [1926; 2763] vs 2275 [2043; 2767]; *p* < 0,001), and (2301 [1894; 2773] vs 2419 [2024; 2680]; *p* < 0,001)); in carbohydrate intake ((310 [266; 381] vs 361 [319; 423]; *p* < 0,001), (302 [262; 401] vs 356 [303; 450]; *p* < 0,001), and (324 [245; 393] vs 360 [302; 423]; *p* < 0,001)); in sodium intake ((3480 [2272; 4698] vs 3717 [2659; 4929]; *p* < 0,001), (3450 [2463; 4603] vs 3689 [2715; 4892]; *p* < 0,001), and (3741 [2529; 4980] vs 3989 [2963; 5210]; *p* < 0,001)); in calcium intake ((458 [373; 609] vs 525 [427; 666]; *p* < 0,001), (497 [403; 616] vs 568 [468; 705]; *p* < 0,001), and (475 [373; 638] vs 531 [452; 690]; *p* < 0,001)) as well as a significant decrease in protein intake ((73 [51; 95] vs 65 [59; 75]; *p*=0.003), (73 [57; 86] vs 65 [58; 74]; *p* < 0.001), and (75 [56; 91] vs 68 [61; 75]; *p* < 0.001)) and lipid intake ((69 [56; 95] vs 62 [58; 80]; *p* < 0.001), (78 [58; 94] vs 68 [58; 83]; *p* < 0.001), and (84 [ 61; 99] vs 72 [59; 89]; *p* < 0.001)), respectively. SFA showed a nonsignificant increase for the age group [18; 25 years], (24 [19, 33] vs 27 [22, 34]; *p*=0,3), and a nonsignificant decrease for subjects aged 40 years and older, (30 [22; 38] vs 28 [22; 38], *p*=0,4), while no change was observed for age group ]26; 40 years[, (28 [21, 36] vs 28 [22, 35]; *p*=0,8). Sucrose recorded an insignificant increase for the age group [18; 25 years], (17 [12, 21] vs 7.5 [14, 20]; *p*=0.3), and for the group 40 years and more, (17 [13, 21] vs 18 [14, 21]; *p*=0.2), while no variation was observed for the age group ]26; 40 years[, (18 [12, 22] vs 18 [13, 22]; *p*=0.9).

## 4. Discussion

This study revealed a considerable increase in household spending on food during Ramadan, regardless of the household standard of living, exceeding by 50% that of the other months of the year. This increase is larger than that reported by the Moroccan National Household Consumption and Expenditure Survey of 2014, estimated at 37%, with significant differences according to the standard of living of households. The same survey showed that the food products which contribute the most to this additional expenditure are fruit, meat, cereals, and milk and milk products, which has an impact on the evolution of consumer prices [3].

The nutritional risk, assessed by the comparison of DNI with RNI [24, 25], revealed that the nutritional intakes were inadequate both before and during Ramadan. It is also evident that malnutrition by deficiency or by nutritional excess is strongly associated with the occurrence or worsening of deficiency diseases or chronic diseases [11, 12, 26–28]. In the population studied, this nutritional risk is aggravated by the effect of Ramadan which could accentuate the food imbalance. Indeed, the study revealed a significant increase in daily energy intake during Ramadan exceeding the RNI by 18%. This result is in line with those of the studies carried out on the Moroccan and Tunisian populations [4, 5, 9], but in disagreement with other studies that reported either a decrease [6] or comparable energy inputs before and during Ramadan among other Tunisian populations [29, 30]. As the increase in energy intake depends on the consumption of high-energy density foods associated with insufficient physical activity, it would be wise to limit the intake of carbohydrates, fat, and free sugars to avoid weight gain [26, 27, 31–33]. In addition, the increase in energy intake could be explained by a high consumption of food sources of energy to cover the needs for the body and activities during fasting. In fact, carbohydrate intake increased during Ramadan, exceeding the RNI by 38%. This finding is consistent with studies conducted in Moroccan and Saudi populations [4, 34] but disagrees with a study conducted in a Tunisian population [5]. The increase in carbohydrate intake revealed by the studied population could be due to a greater consumption of cereal products, basic foods in the Maghreb [9, 35], which are experiencing a greater demand by the Moroccan population during Ramadan [3].

Similarly, for the Tunisian population [6], the intake of simple sugars during Ramadan in this study far exceeded the recommendations (80%). This increase could be due to the abundant consumption of traditional Moroccan preparations rich in simple sugars, in particular, chebakia, sellou, and sweet briwates specific to the month of Ramadan [35, 36].

Protein intake decreased slightly during Ramadan while remaining high by 30% higher than the RNI. Other studies have reported that protein intake increases during Ramadan among Moroccan, Tunisian, and Saudi populations [4, 5, 9, 34]. The decrease shown here in our study could be explained by a lack of protein food consumption [9] despite the greater demand that this food experienced by the Moroccan population during Ramadan [3].

In addition, unlike other studies on Tunisian and Saudi populations [5, 9, 34], the lipid intake decreased in our sample during Ramadan, reaching 4% less compared to the RNI. The variation in lipid intake could be attributed to the relatively high consumption of fats and frying during this sacred month [36]. Calcium intake increased during Ramadan but remained lower (-33%) than recommended. This improvement in calcium intake is linked to a consumption of milk and its derivatives, which is one of the demands in the Moroccan population especially greater during this month [3].

Sodium, the intake of which also increased significantly during Ramadan (64%) compared to the recommendations, could be explained by the high consumption of cereal-based preparations in the 1st meal of break of the fast (ftour), in particular, the bread reported to be the vehicle of high quantities of sodium chloride [37] as well as other products such as quiches, stuffed bread, briks, and salted briwates.

The variations in energy, protein, carbohydrate, and lipid intake with the age factor are in agreement with the results of the literature [9]. However, the increase in SFA in the youngest [24–31] and their decrease in subjects over the age of 40 could be linked to a higher consumption of fatty preparations and fried foods by the young. In addition, calcium intake did not change with age in this study, as compared to other studies that reported increase in the population aged over 60 [8] or in a population aged 19 to 25 [9].

In general, the variations in food consumption described in this study could be explained by the change in eating behavior, fostered by an atmosphere of festive solidarity and family grouping during the 30 days of the holy month of Ramadan [29, 36].

### 4.1. Limitations of the Study

- The results of this study, although relevant, are not representative of the 11 other regions of the country or even of the studied region. Extending this study to a larger representative sample would be wise to generalize the results obtained.
- Another limitation is that the study did not compare the composition of the food basket before and during Ramadan. Carrying out a qualitative study would make it possible to formulate practical and appropriate dietetic advice, likely to promote better health of the population.

## 5. Conclusion

This study assessed the nutritional risky behaviors and examined the variation in food expenditure and dietary intake of Moroccan households during Ramadan. The results obtained revealed that food expenditure is likely to increase during Ramadan, proving that food consumption increases in quantity and quality during this sacred month. In addition, some dietary behaviors adopted during Ramadan can improve the population nutritional status, like the revealed increase in calcium intake, while others behaviors are harmful and in favor of the development of overnutrition diseases and chronic diseases. Indeed, the study revealed significant increase in intakes of energy, carbohydrate, sucrose, and sodium and a significant decrease in protein intake and lipid intake. All these results justify the need to carry out mass educational actions in Moroccan population to adopt healthy eating practices during Ramadan.

## Figures and Tables

**Table 1:** Sociodemographic and socioeconomic characteristics (*n* = 340).

Characteristics	Values
Age groups^*∗*^	
[18–25 years]	66 (19%)
]26–40 years[	111 (33%)
≥40 years	163 (48%)

Gender^*∗*^	
Male	177 (52%)
Female	163 (48%)

Area of residence^*∗*^	
Urban	240 (70.6%)
Rural	100 (29.4%)

Standard of living^*∗*^	
Low	116 (34%)
Middle	137 (40%)
High	87 (26%)

Food expenditure^*∗∗*^	
Before Ramadan (MAD)	1600 [1000; 2000]
During Ramadan (MAD)	2500 [2000; 3000]
Taux de variation pendant Ramadan (%)	50 [22; 67]

^*∗*^Values are expressed as number (*p*%); ^*∗∗*^values are expressed as median and quartile; MAD = Moroccan dirham (official currency of Morocco).

**Table 2:** Food expenditure before and during Ramadan according to standard of living (*n* = 340).

Food expenditure	Standard of living			*P* ^*∗∗*^
	Low	Middle	High	
Before Ramadan (MAD)^*∗*^	1000 [1000; 1200]	2000 [1500; 2000]	3000 [2500; 3000]	<0.001
During Ramadan (MAD)^*∗*^	1500 [1000; 2000]	2500 [2000; 3000]	4000 [3000; 5000]	<0.001
Rate of variation (%)^*∗*^	50 [20; 100]	39 [25; 52]	40 [20; 67]	0.35

^*∗*^Values are expressed as median and quartile; ^*∗∗*^test of Kruskal–Wallis (the mean difference is significant at the 0.05 level); MAD = Moroccan dirham (official currency of Morocco).

**Table 3:** Rate of variation in DNI according to RNI (*n* = 340).

Characteristics	Before Ramadan			During Ramadan		
	DNI^*∗*^	RNI	Variation (%)	DNI^*∗*^	RNI	Variation (%)
Energy (kcal/day)	2247 [1895; 2745]	2000	12	2367 [2040; 2700]	2000	18
Protein (g/day)	74 [55; 89]	50	48	65 [60; 75]	50	30
Carbohydrate (g/day)	312 [253; 391]	260	20	360 [303; 430]	260	38
Sucrose (% of TEI)	17 [12, 21]	10	70	18 [14, 21]	10	80
Lipids (g/day)	78 [59; 97]	70	11	67 [58; 85]	70	−4
SFA (g/day)	28 [22, 37]	20	40	28 [23, 37]	20	40
Sodium (mg/day)	3688 [2452; 4873]	2400	54	3928 [2772; 5121]	2400	64
Calcium (mg/day)	478 [377; 623]	800	−40	540 [452; 689]	800	−33

^*∗*^Values are expressed as median and quartile; TEI = total energy intake; SFA = saturated fatty acid; DNI = daily nutritional intake; RNI = recommended nutrient intake.

**Table 4:** Nutritional intake comparison before and during Ramadan (*n* = 340).

Characteristics	DNI before Ramadan	DNI during Ramadan	*P* ^*∗∗*^
Energy^*∗*^ (kcal/day)	2247 [1895; 2745]	2367 [2040; 2700]	<0.001
Proteins^*∗*^ (g/day)	74 [55; 89]	65 [60; 75]	<0.001
Carbohydrates^*∗*^ (g/day)	312 [253; 391]	360 [303; 430]	<0.001
Sucrose^*∗*^ (% TEI)	17 [12, 21]	18 [14, 21]	0,02
Lipids^*∗*^ (g/day)	78 [59; 97]	67 [58; 85]	<0.001
SFA^*∗*^ (g/day)	28 [22, 37]	28 [23, 37]	0.86
Sodium^*∗*^ (mg/day)	3688 [2452; 4873]	3928 [2772; 5121]	<0.001
Calcium^*∗*^ (mg/day)	478 [377; 623]	540 [452; 689]	<0.001

^*∗*^Values are expressed as median and quartile; ^*∗∗*^Wilcoxon test for paired series (the mean difference is significant at the 0.05 level); TEI = total energy intake; SFA = saturated fatty acid; DNI = daily nutritional intake.

**Table 5:** Comparison of nutritional intake before and during Ramadan according to age groups (*n* = 340).

Age groups	Energy (Kcal/day)	Proteins (g/day)	Carbohydrates (g/day)	Sucrose (% of TEI)	Lipids (g/hour)	SFA (g/day)	Sodium (mg/day)	Calcium (mg/day)
[18–25] *years*								
Before Ramadan^*∗*^	2203 [1862; 2699]	73 [51; 95]	310 [266; 381]	17 [12, 21]	69 [56; 95]	24 [19, 33]	3480 [2272; 4698]	458 [373; 609]
During Ramadan^*∗*^	2328 [2037; 2689]	65 [59; 75]	361 [319; 423]	17.5 [14, 20]	62 [58; 80]	27 [22, 34]	3717 [2659; 4929]	525 [427; 666]
*P* ^*∗∗*^	0.001	0.003	<0.001	0.3	<0.001	0.3	<0.001	<0.001

*]26–40[ years*								
Before Ramadan^*∗*^	2178 [1926; 2763]	73 [57; 86]	302 [262; 401]	18 [12, 22]	78 [58; 94]	28 [21, 36]	3450 [2463; 4603]	497 [403; 616]
During Ramadan^*∗*^	2275 [2043; 2767]	65 [58; 74]	356 [303; 450]	18 [13, 22]	68 [58; 83]	28 [22, 35]	3689 [2715; 4892]	568 [468; 705]
*P* ^*∗∗*^	<0.001	<0.001	<0.001	0.09	<0,001	0.8	<0.001	<0.001

*≥40 years*								
Before Ramadan^*∗*^	2301 [1894; 2773]	75 [56; 91]	324 [245; 393]	17 [13, 21]	84 [61; 99]	30 [22; 38]	3741 [2529; 4980]	475 [373; 638]
During Ramadan^*∗*^	2419 [2024; 2680]	68 [61; 75]	360 [302; 423]	18 [14, 21]	72 [59; 89]	28 [22; 38]	3989[2963; 5210]	531 [452; 690]
*P* ^*∗∗*^	<0.001	<0.001	<0.001	0.2	<0.001	0.4	<0.001	<0.001

^*∗*^Values are expressed as median and quartile; ^∗∗^test of Wilcoxon (the mean difference is significant at the 0.05 level); TEI = total energy intake; SFA = saturated fatty acid.

## Data Availability

The survey data used to support the findings of this study are available from the corresponding author upon request.

## Abbreviations

DNI: Daily nutritional intake
g: Gram
Kcal: Kilocalorie
MAD: Moroccan dirham (official currency of Morocco)
mg: Milligram
RNI: Recommended nutrient intake
RSK: Rabat-Salé-Kenitra
SFA: Saturated fatty acid
SPSS: Statistical Package for the Social Sciences
TEI: Total energy intake
Vs: Versus.
