# Corrigendum to “Food Expenditure and Food Consumption before and during Ramadan in Moroccan Households”

**DOI:** 10.1155/2022/9839623

**Published:** 2022-03-18

**Authors:** Imane Barakat, Hamid Chamlal, Sanaa El jamal, Mohammed Elayachi, Rekia Belahsen

**Affiliations:** Laboratory of Biotechnology Biochemistry and Nutrition, School of Sciences, Chouaib Doukkali University, El Jadida 24000, Morocco

In the article titled “Food Expenditure and Food Consumption before and during Ramadan in Moroccan Households” [1], there were errors in the References section. The updated references are shown in the following.

[1] J. Ramadan, G. Telahoun, N. S. Al-Zaid, and M. Barac-Nieto, “Responses to exercise, fluid, and energy balances during Ramadan in sedentary and active males,” Nutrition, vol. 15, no. 10, pp. 735–739, 1999.

[21] G. Dufourny, K. Elmoumni, and E. Maimouni, “Aliments et préparations typiques de la population Marocaine, outil pour estimer la consommation alimentaire,” Centre d'Information et de Recherche sur les Intol´erances et l'Hygiène Alimentaires (CIRIHA), p. 158, 2008.
